# Tactile sensitivity alters textile touch perception

**DOI:** 10.1371/journal.pone.0308957

**Published:** 2024-09-18

**Authors:** Sunidhi Mehta, Ida Holásková, Matthew Walker

**Affiliations:** 1 Davis College of Agriculture, Natural Resources and Design, West Virginia University, Morgantown, West Virginia, United States of America; 2 Office of Statistics and Data Analytics, West Virginia Agriculture and Forestry Experiment Station Davis College of Agriculture, Natural Resources and Design, West Virginia University, Morgantown, West Virginia, United States of America; The University of Electro-Communications, JAPAN

## Abstract

Tactility plays a crucial role in our interactions with the physical world including our ability to differentiate textile textures and their associated comfort. There is an increasing focus on digitally interactive haptic experiences enabling consumers to feel virtual objects realistically. This could revolutionize how we experience textiles in e-commerce platform, virtual and augmented reality, and shape the future of textiles in the metaverse. In this study, we examined the impact of tactile sensitivity on touch perception of a large nonhomogeneous sample of 22 textile swatches. The tactile sensitivity was studied using four factors: assessors’ “subject-matter expertise”, “frequency of performing handiwork”, “frequency of working with textiles”, and “familiarity of textile textures”. The participants noted their tactile assessment of eight touch attributes of textile swatches on a 5-point Likert scale. Through predictive modeling, we analyzed the effect of tactile sensitivity on participants’ tactile assessment scores. Our key findings revealed that participants’ tactile sensitivity significantly influenced their perception of the textile textures. Notably, the “frequency of working with textiles” had the most substantial impact on participants’ tactile ratings followed by their familiarity with textile textures. Interestingly, the perceptual differences of isotropy attribute were significant in all the cases. Overall, there was no significant difference in the tactile ratings between textile experts and non-experts, except for nine occurrences, four of which were related to perceptual differences in roughness of the woven fabrics. Conversely, the two groups had no statistically significant differences at all in their perceptions of hairiness, scratchiness, and uniformity.

## Introduction

Tactility is the capacity to perceive and interpret the physical sensations of a material or its surface through the sense of touch [1]. It plays a crucial role in our interactions with the physical world, including our ability to perceive and differentiate various textile textures [2]. When it comes to perceiving textures, the sense of touch relies on specialized nerve endings in our skin called mechanoreceptors [3]. The two main types of mechanoreceptors involved in tactile perception are Meissner’s corpuscles and Pacinian corpuscles [3]. Meissner’s corpuscles are mechanoreceptors located close to the skin surface, particularly in areas such as the fingertips, palms, and soles of the feet. They are sensitive to light touch and are responsible for perceiving fine details and textures of the material in contact with our skin [2,3]. On the other hand, Pacinian corpuscles are found deeper within the skin and are more responsive to vibrations and deep pressure. The information gathered by these receptors is transmitted as electrical signals through nerve fibers to the brain [4]. The brain plays a vital role in the processing and interpretation of these signals to create the perception of textile textures. The primary somatosensory cortex, located in the parietal lobe of the brain, receives and analyzes tactile information [5,6]. Different regions within this cortex are responsible for processing specific aspects of touch, such as texture, pressure, and temperature [7,8].

In the field of textiles, tactility is often used to perceive the fabric texture and the associated perceived comfort of garments. Textile tactility has been an area of great interest among researchers and in the apparel industry for several decades. This became an important question for the research community in the early 2000s with the escalating popularity of fashion e-commerce. From a consumer standpoint, one of the biggest challenges of online apparel shopping is the lack of tactility and consumer’s inability to perceive the associated comfort and feel of the garment [9] leaving them with post-purchase cognitive dissonance. To better understand textile touch perception, several researchers [8–13] have conducted quantitative and qualitative studies, including the latest instrument in the field called the Fabric Touch Tester (FTT). FTT measures the thermal effects, compressive capability, mechanical bending, fabric shearing, and surface friction using a single sample. Although these properties determine touch perception [13–17] they do not adequately capture the complex interactions between skin and textile surface involved in human tactile perception [17].

Textile tactility research began in the 1930s, with the textile engineering community’s ever-growing interest in measuring fabric parameters to quantify its drape and handle [18]. For nearly a century-old research area, scientists are still developing comparable methods of interacting with virtual textiles the way consumers do in a physical environment [5–9,18,19]. The ability to simulate the sensation of touching digital textiles could revolutionize the retail experience of 3D digital products, specifically online apparel shopping [20,21]. We predict that artificial intelligence (AI) and machine learning (ML) will play significant roles in achieving this goal in the textile and apparel industry. Similar to virtual apps for garment fit, AI algorithms for immersive haptic experiences can provide consumers with accurate textural information. Consequently, they can make better purchase decisions and have less post-purchase cognitive dissonance. Moreover, haptic technology can facilitate a virtual try-on experience, enabling consumers to assess the fit, garment feel, comfort, and functionality.

Additionally, as the textile industry moves rapidly towards achieving Industry 4.0, the focus on adopting ML techniques is witnessing unprecedented growth for various applications across the industry [22]. However, currently the biggest hurdle in accomplishing these goals is the lack of scientific data for ML applications [22]. Keeping this research gap in mind, we designed this study to develop a deeper understanding of textile tactility through human assessment.

Through a novel approach, we studied the impact of tactile sensitivity on human touch perception of textile surfaces via predictive modeling and investigated the interrelationships of the touch attributes that cumulatively form the perception of textile textures. We anticipate that the findings of this study will be beneficial for researchers developing ML-based assessments of fabrics’ mechanical properties, design of haptic devices, augmented and virtual reality, and e-healthcare applications, including for people with tactile hypo-or-hypersensitivity. E-commerce textile retailers can also use this information to better present their merchandise in the digital environment and create an immersive experience for their consumers. Additionally, using this information, brands can create AI-based personal shopping experiences for their consumers. For instance, AI algorithms can analyze customer preferences and purchase history to recommend products with similar fabric types and textures they have liked before. This will lower the post-purchase cognitive dissonance of textile products related to a lack of tactile experience in e-commerce shopping.

## Materials and methods

### Study ethics approval

This study was approved by the institutional review board (IRB) of West Virginia University, and all methods used in this study were in accordance with the IRB guidelines and regulations. All participants provided written informed consent for voluntary participation in this study. In addition, all researchers involved in the study completed the CITI ethics training prior to the beginning of the study.

### Participant recruitment procedure

#### Non-expert participants (NEP)

All the non-expert participants of this study were undergraduate and graduate students enrolled in various (STEM and non-STEM) programs at a Research 1 University located in the mid-Atlantic region of the United States of America. We advertised the study via mass email and posted study flyers at all buildings of the university to reach a wide study sample. A total of 34 prospective participants expressed interest and participated in a pre-experimental electronic survey. Out of these, a total of 20 participants were selected to take part in the study. Owing to the nature of the study, we wanted to ensure that the participants did not have any outlying tactile impairments or conditions. Therefore, individuals with any such condition were excluded from the study. Further details of the pre-experimental survey are discussed in the following section.

#### Textile Experts (TE)

A panel of five university professors (ages between 38–45 years) was invited to provide their expert assessment of eight touch attributes of the 22 textile swatches as reference scores. All the experts have 7+ years of experience teaching in the Apparel and Textile degree programs in the USA and hold a terminal degree in the field. We acknowledge that the small sample size of the TE group may limit the generalizability of the findings of this study. However, we anticipated and observed lesser variability in the tactile assessment scores of the TE group due to the group’s cohesiveness, as they share similar educational background, experience, and expertise in textiles. We confirmed this by using the Levene test to measure the homogeneity of variance between the TE and NEP scores, ensuring that their variances are comparable despite the differing sample sizes. Further, the power of the statistical test for two independent sample means using n = 5 for TE and n = 20 for NEP, alpha 0.05, and delta (difference in Likert scale to detect) of 2, was computed.

### Pre-experimental survey

We collected the demographic data of all the interested participants in a pre-experimental electronic survey. The survey included demographic and pre-screening questions related to the study. A total of 20 qualifying participants (15 female and 5 male; 12 Caucasian; 5 Hispanic; 2 Asian; and 1 mixed-race) aged between 18 to 35 years were selected to participate in the study. In the case of educational background, 12 were pursuing education in STEM-related fields, while the remaining eight participants had a non-STEM educational background. When asked about hand dominance, 17 indicated right-hand dominance and 3 indicated left-hand dominance. For consistency, all participants were only allowed to use the index finger of their dominant hand to perceive the fabric texture [21].

To ensure the assessment of tactile sensitivity, we asked prospective participants several pre-screening questions during the pre-experimental survey. These questions were inspired by the study conducted by Degrean et al. [21]. All selected participants confirmed that they did not suffer from any tactility impairment such as arthritis and/or hypoesthesia. Furthermore, they also confirmed that they had not undergone any haptic therapy in the past five years. Finally, the participants also provided Likert scale ratings on their level of engagement/working with textiles (H:5, M:5, L:10), familiarity with textile textures (H:10, M:5, L:5), along with the frequency of handiwork (excluding textiles) performed (H = 8, M = 8, L = 4). To our knowledge, these three factors have not been previously explored in a published research study in the field of textile touch perception. Therefore, we hypothesized that they can be contributing factors in the tactile assessment of textile swatches and presented our analysis.

### Textile swatches

**Fig 1:**
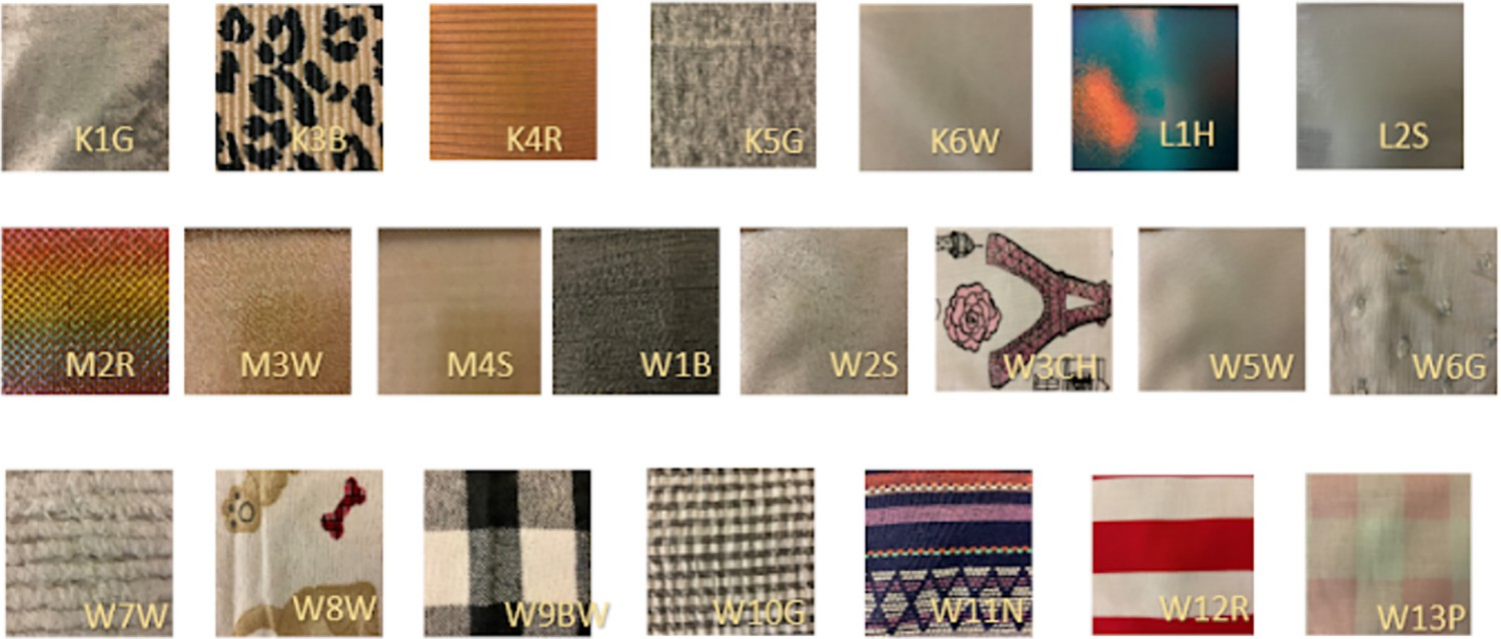
The twenty-two textile swatches and their respective codes were presented during the experiment for the tactile evaluation. The top row has knitted, and laminated textile swatches followed by mesh and woven swatches in the middle row that continue through the third row.

**Fig 2:**
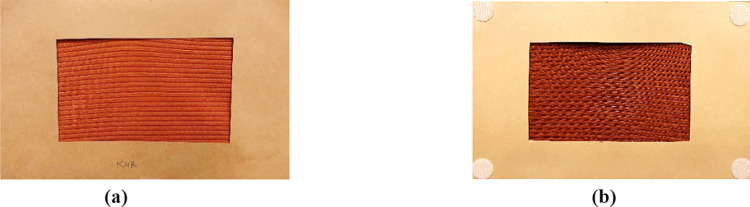
An example of a mounted textile swatch presented to the study participants. The fabric swatch was securely mounted between two thick sheets of paper to prevent it from wrinkling which could affect the tactile perception. The right side surface of the fabric as shown in Fig 2A was tactilely perceived by the participants while Fig 2B shows the backside of the swatch with velcro hook dots glued at the four corners of the swatch. This allowed each swatch to be placed at a precise location on the table and also prevented the swatch to move and slide.

We curated a set of 22 textile swatches, representing a wide range of textile textures used in the apparel industry. The textile swatches had varying fiber compositions (natural, blended, and synthetic), fabric construction (knitted, woven, mesh, and laminated), and weight. The textile swatches along with their respective codes are presented in Fig 1. For consistency, we cut all textile swatches in the dimensions of 8 times 5 inches, mounted between a frame of two hard paper sheets to prevent the fabrics from folding and wrinkling during the tactility assessment (Fig 2). The total exposed dimensions of the fabric in all the samples were 5 times 3 inches. This approach was different from previous studies [8,9,14,15,17,18], where participants were allowed to squeeze textile swatches with their hands. Although the action of squeezing the fabric with hands can be used to perceive the texture of physical textiles, it is not feasible to perform this action in the digital representation of textiles during online shopping. In the digital representation of a textile product, the fabric appears to be flat, and we wanted to follow the same presentation style for this study. Moreover, wrinkles and folds resulting from squeezing the textile swatch can alter the perception of the touch attributes investigated in this study, such as uniformity, isotropy, bumpiness, and hardness.

### Selection of eight touch attributes

To evaluate the tactile feedback of textile swatches, we used a psychophysical experimental approach grounded in the literature [17,21,22–26]. We presented 22 textile swatches consisting of various textures and fiber compositions to 20 non-expert participants. They were asked to perceive each textile swatch for its tactile impressions in terms of hardness, roughness, bumpiness, stickiness, scratchiness, hairiness, uniformity, and degree of isotropic surface texture on a 5-point Likert scale. One indicates a low assessment and five indicates a high assessment of the respective attribute. We chose hardness, roughness, and stickiness because they are considered the basis of tactile exploration models [23–26]. The inclusion of bumpiness, scratchiness, and isotropy was motivated by [25], whereas hairiness and uniformity were motivated by the fact that our samples were limited to textiles. The tiny fibrils are inherent to textile surfaces, which gives them a feeling of hairiness that correlates with the isotropy attribute. This addresses the directionality given by sensing fibrils and could influence these attributes, making them important attributes for consideration in this study. The inclusion of these eight touch attributes in tactile assessment of textile material is another novel aspect of our study.

### Defining the eight touch attributes

**Rough/Smooth**: Fabric roughness relates to a multidimensional sensation of fabric’s texture including the spatial variation of surface protrusions and is the opposite of “smoothness”. It refers to the texture of a surface characterized by fine, small-scale irregularities [27]. These irregularities create friction or resistance when touched or moved across.

**Hard/Soft**: Fabric softness property is considered the opposite of hardness and is related to the properties of compression and flexibility [28].

**Bumpy/Flat**: Bumpiness refers to the presence of noticeable protuberances or bumps on a surface. It is typically associated with variations in height or elevation on a relatively larger scale [23]. One of the major differences between bumpiness and roughness is that the former feels on a macroscale while the latter is a microscale perception of a surface texture.

**Sticky/Slippery:** For the sake of this study, we defined stickiness as the property of feeling clingy and adhesive, while, slippery is the opposite of sticky. An example of a highly sticky surface is velcro tape.

**Scratchy/Slick:** We defined scratchiness as the quality or condition of being abrasive. For instance, pure wool fabric can sometimes feel scratchy on the skin causing itching and irritation. Several studies found a positive relationship between roughness and scratchiness [23,25].

**Hairy/Shorn:** Surface hairiness refers to the presence of fine, hair-like projections on the surface of a textile material. This property gives a fuzzy surface to the fabrics. The textile surfaces feel hairy due to the presence of tiny fibrils in their construction. Shearling, fur, and plush are examples of hairy fabric surfaces.

**Uniform/Irregular:** Textile surface uniformity refers to the consistency and evenness of the surface properties of a fabric. This could include the absence of any protrusions, surface variations, and/or irregularities. One of the major differences between the attributes of “uniformity” and “isotropic” is that uniformity corresponds to the homogeneous texture while isotropic corresponds to uniformity of texture in all directions.

**Isotropic/anisotropic:** Inspired by Degraen et al [21] we included isotropy attribute in our study. In context to this study, we defined surface isotropy as the uniformity of textural elements of a fabric surface in all directions. While traditional woven and knitted fabrics tend to have anisotropic properties due to the directional alignment of yarns, special techniques like balanced weaves or bi-axial knits can enhance isotropy. Nonwovens, made by bonding or interlocking fibers through mechanical, thermal, or chemical means, can exhibit isotropic properties because the fibers are randomly oriented. Additionally, laminated fabrics have a higher isotropic surface feel compared to traditional woven and knitted fabrics with unidirectional structures.

### Experimental setup and data collection

Upon arrival, each participant was briefed on the expected tasks and experimental setup by the researcher. They provided their written consent for voluntary participation in the study. We also described the eight touch attributes and gave precise instructions on how to perceive the fabric texture during the experiment. Participants were instructed to perceive the fabric using only the index finger of their dominant hand in three possible directions. As the study examined the "isotropy" attribute of texture directionality, we asked participants to perceive the texture by moving their fingers horizontally, vertically, or in circular motions on the fabric surface. Additionally, participants were asked to maintain steady finger pressure and movement, avoiding excessive pressure and rapid movements. The pressure, speed, and direction of finger movement can significantly influence the perception of fabric texture; thus, consistency in these factors was crucial as participants evaluated all textile samples [29,30].

**Fig 3:**
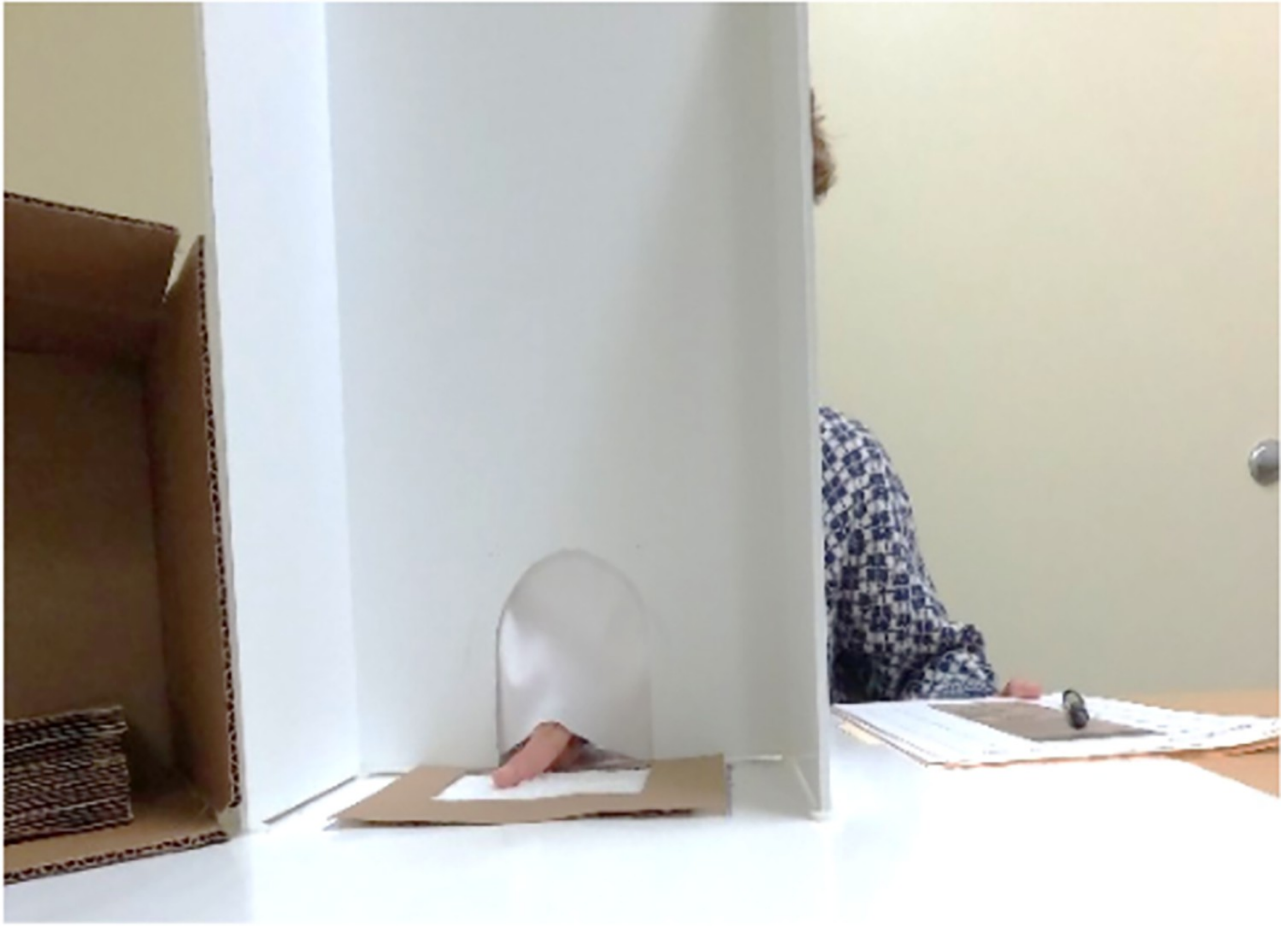
The researcher-side view shows participant tactilely exploring the textile swatch behind the screen with their dominant index finger. The participant provided informed consent for their photograph to be published in an online open-access publication.

The participant was asked to sit comfortably on a chair behind the screen. The index finger of the participant’s dominant hand was cleaned with isopropyl alcohol wipes to remove any traces of dust, grime, or sweat that could subtly alter the touch perception of the textile swatch surface. They were also provided with a printed list of all textile swatch pictures (Fig 3). As a next step, the researcher placed one of the textile swatches at the fixed location behind the screen. The participants were then asked to slide in their hand and perceive the texture of the presented swatch with their dominant hand’s index finger without looking at it (Fig 3).

To control the study duration and capture participants’ first impressions of the fabric texture, they were allowed to perceive each swatch tactilely for only 20 seconds. The presentation order of the swatches was randomized for each participant to overcome fatigue associated with the order effect. The participants perceived a large number of textile swatches in this study, therefore, some physical and mental fatigue may be associated with the expected task. Hence, randomizing the order in which the swatches were presented to each participant was important for removing the order effect. In addition, breaks were provided to each participant upon request. After perceiving a swatch, the participant was asked to rate the swatch for eight tactile attributes on a 5-point Likert scale. The participants verbalized their responses, and the researcher noted the responses electronically for the corresponding textile swatch. At the end of the survey, the researcher replaced the swatch with another until the responses for all 22 swatches were gathered. The following questions were sequentially asked by the researcher to each participant.

Q1: How **hard** is the fabric surface? (1 meaning extremely soft and 5 meaning extremely hard)

Q2: How **rough** is the fabric surface? (1 meaning extremely smooth and 5 meaning extremely rough)

Q3: How **bumpy** is the fabric surface? (1 meaning extremely flat and 5 meaning extremely bumpy)

Q4: How **sticky** is the fabric surface? (1 meaning extremely slippery and 5 meaning extremely sticky)

Q5: How **scratchy** is the fabric surface? (1 meaning extremely slick and 5 meaning extremely scratchy)

Q6: How **hairy** is the fabric surface? (1 meaning extremely shorn and 5 meaning extremely Hairy/Shaggy)

Q7: How **uniform** is the fabric surface? (1 meaning extremely irregular and 5 meaning extremely uniform)

Q8: How **isotropic** is the fabric surface? (1 meaning extremely anisotropic and 5 meaning extremely isotropic)

### Statistical analyses

The distribution of Likert scores was examined for each textile swatch and touch attribute using the Shapiro-Wilk W test if the scores were normally distributed, in addition to the Levene test to assess the homogeneity of variance between the groups. The lack of normality was detected in the majority of groups. Therefore, the nonparametric procedures were utilized in the study. The homogeneity of variance between TE and NEP groups was done using Levene test two-ways: firstly by the eight touch attributes and secondly by the individual fabrics (22 swatches times 8 attributes = 176). In addition, the power of the test was calculated for two independent sample means for the eight touch attributes.

The median scores comparison between textile experts and non-expert participants for each textile swatch across all the eight touch attributes was done using the nonparametric Wilcoxon signed-rank test. Each test (for each fabric and touch attribute) included n = 25 observations (20 NEP and 5 TE). The significance criterion alpha for all statistical tests was 0.05. Due to the large number of tests performed (176; 22 textile swatches times 8 touch attributes) and associated increased chance of statistical Type I error (false positive detection of differences) Benjamini-Hochberg (BH) [31] adjustment with 0.1 false discovery rate (FDR) was utilized. The *Q-value* is the “new”, more conservative *p-value* after the BH adjustment.

The correlation of Likert scores from both the textile experts and non-expert participants between the pairs of the eight touch attributes was estimated using nonparametric Spearman’s Rho, where fabrics were grouped into four groups based on the fabric construction (knitted, laminated, mesh, and woven), for a total of 28 times 4 = 112 tests. For the same reason as above, the p-values from 112 correlation analyses were ranked and the BH procedure with the 0.1 FDR was applied. Correlations were evaluated on n = 125 observations for knitted (5 fabrics times 25 assessors), n = 50 for laminated (2 fabrics times 25 assessors), n = 75 for mesh (3 fabrics times 25 assessors), and n = 300 for woven (12 fabrics times 25).

Experimental hypotheses that the “frequency of the precise handiwork”, “frequency of working with textiles”, “familiarity of textile textures”, and the fabric construction are associated with the scores given to individual textile swatches across all the eight touch attributes were tested using multiple ordinal logistic regression (OLR) models. The response variable (Y)- the scores of fabric touch attributes (ordinal Likert scale 1 to 5) had five ordinal levels. The independent variables (X) included the Likert scores of variables obtained from the survey of the non-expert subjects for frequency of the precise handiwork, frequency of working with textiles, and familiarity with textile textures, which were each grouped into three categories, low (1 and 2), medium (3) and high (4 and 5); and also included four categories of fabric construction (knit, laminated, mesh and woven). The OLR model for each attribute utilized n = 440 observations (22 fabrics times 20 assessors). The OLR takes into consideration the order (smallest to largest) nature of the Likert scale response and has the flexibility of using either continuous or categorical independent variables. When the response has an order (eg.: extremely soft = 1 to extremely hard = 5), the cumulative probabilities of the response levels are computed as cumulative logits using the proportional odds model [32,33]. In multiple OLR models for each of the eight attributes, each model utilized all four X variables. The OLR model evaluated the probability of an equal or smaller response, *Y≤ k*, was compared with the probability of a larger response, *Y>k*, where *k* is the rank of ordinal response categories (*K* = 1 to 5). For example, with five ordinal response categories for hardness, there were four (*K*-1) cumulative logits (Log of ratio of two odds): **1)** Log of odds of scoring the fabric as extreme soft against all other levels, **2)** Log of odds of extremely soft and moderately soft against all remaining higher levels, **3)** Log of extremely soft, moderately soft and neutral against moderately hard and extremely hard, and **4)** Log of the probability that hardness was equal to four or less, divided by the odds that the response is the hardest, given the combination of the four independent variables.

The generalized model in the logit format was

$$h_{k}(x)=Ln[P(Y\leq k|x)/(P(Y>k|x)]$$ (Eq 1)

where:

h_k_(**x**) = proportional odds of *k*, *k* = index of the four possible logits based on the five ordinal response levels

**x** = matrix of the four explanatory variables (X)

*P* = probability

*Y =* the response level (Likert score or the hardness, for instance, predicted)

*k*|**x** = specific level of the response, given the combination of the independent variables X (each X variable number of levels minus 1)

With four logits (intercepts) and nine combinations of the four X variables, considering the degrees of freedom for X variables, (3–1 for frequency of precise handiwork)+(3–1 for frequency of working with textiles)+(3–1 for familiarity with textures)+(4–1 for the fabric construction), there were 13 parameter estimates to make the specific prediction equations for each of the attributes.

The estimate of linear predictor had a general form:

$$g{\left(x\right)}_{k}=\beta_{0k}+x’\beta =\beta_{0k}+x_{1}\beta_{1}+x_{2}\beta_{2}+x_{3}\beta_{3}+x_{4}\beta_{4}$$ (Eq 2)

where:

g(x)_k_ = the linear predictor for each specific logit

**x**’ = the vector of independent variables

*β*_*0k*_ = intercept—specific to each logit

**β** = vector of regression coefficients

x_1_ = level of frequency of the precise handwork

x_2_ = level of frequency of working with textiles

x_3_ = level of familiarity of textile textures

x_4_ = level of fabric construction

β;_1_-β;_4_ = regression parameters, coefficients (slopes) from the parameter table

The fitted value of the ordinal regression model, the actual cumulative probability

$$y=1/(1+\exp(\beta_{0k}‐x’\beta )),\;\mathrm{where}$$ (Eq 3)

y = the predicted probability of scoring the fabric attribute

β;_0k_ = the intercept corresponding to the particular logit, specific *k = K-1*

**x**’**β;** = the linear predictor calculated in Eq 2 (depending on the specific combination on the X variables).

JMP software was used (JMP®, Version Pro 16.0.0, SAS Institute Inc., Cary, NC, Copyright ©2021) for all the analyses. The *p*-values (32) associated with the *LogWorth* of each of the four independent factors for each touch attribute from the ordinal logistic models were also ranked and the final significance was determined after BH with 0.1 FDR. The *LogWorth* statistic is defined as -log10(*p*-value). This transformation adjusts *p*-values to provide an appropriate scale if one would like to use it for graphing and the *p*-values are very small. A value that exceeds 2 is significant at the 0.01 level (because -log10(0.01) = 2). A value that exceeds 1.3 is significant at the 0.05 level (because -log10(0.05) = 1.3). Note that small *p*-values result in high *LogWorth* values and relate to the original *p*-values.

## Results

### Homogeneity of variance between TE and NEP groups

As noted in the methods section, we measured the homogeneity of variance among TE and NEP groups using Levene tests. For the first test, we found five (bumpiness, hairiness, hardness, roughness and uniformity) of the eight attributes (63%) had no difference in variance between experts and non-experts scores (S1 Table). Additionally, for scratchiness and stickiness the variance among experts was smaller than the non-expert participants. Interestingly, the TE group had higher variance for the isotropy attribute compared to the NEP scores. In the case of the second set of comparisons, 94% of the times there was either no difference or lesser variance among the TE scores compared to the NEP scores. The power of statistical tests comparing two independent sample means for all eight attributes was above 61% (S1 Table).

### Tactile assessments of textile experts versus non-expert participants

**Fig 4:**
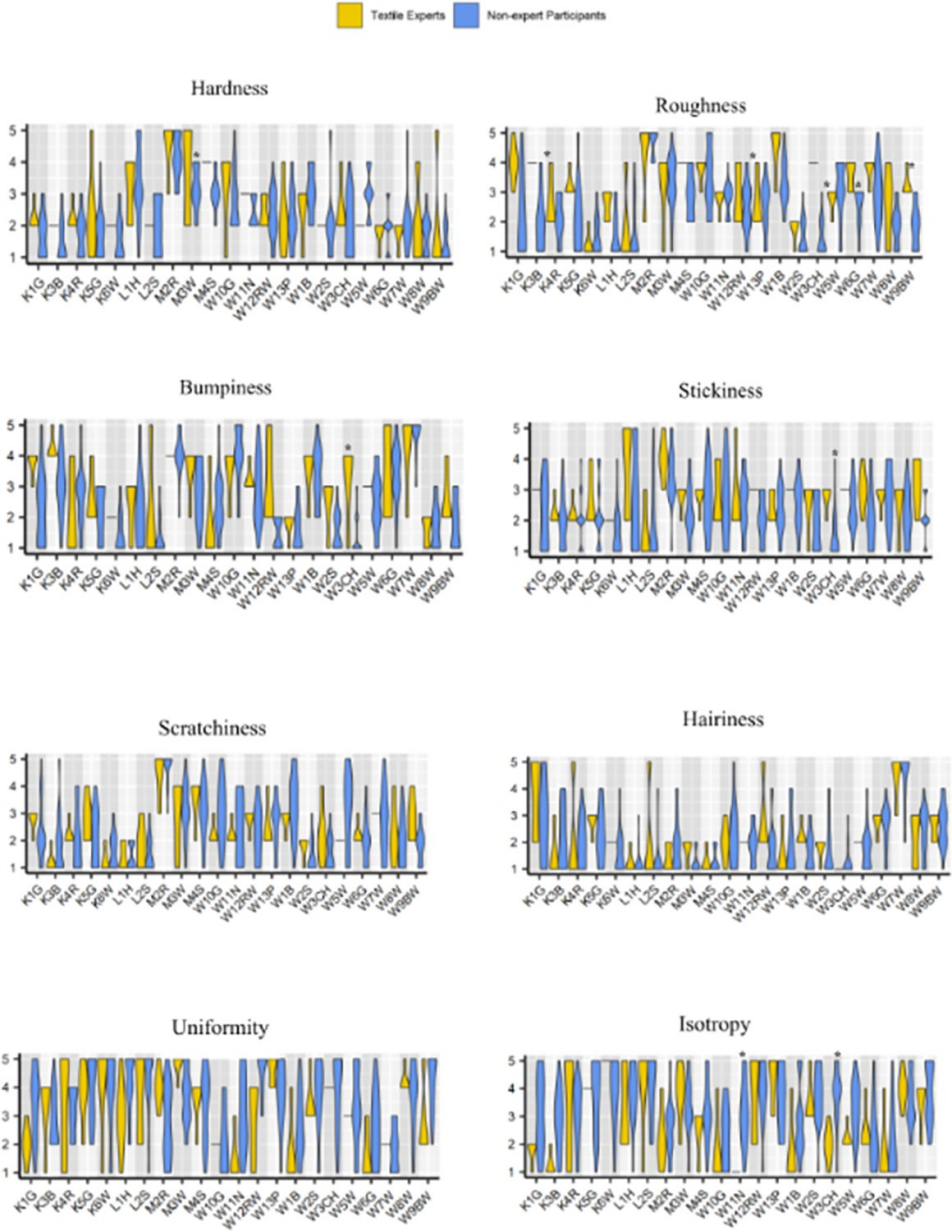
Violin plots comparing textile expert and non-expert participants’ individual ratings of eight touch attributes for all the textile swatches. The X-axis represents fabric code (K = Knitted; L = Laminated; M = Mesh; W = woven textiles) and the Y-axis represents the Likert scores. *Asterisk indicates a significant difference between TE and NEP after BH adjustment with 0.1 FDR.

To compare the tactile assessments by TE and NEP, we conducted the Wilcoxon signed-rank test followed by an adjustment to p-values using BH correction (with FDR of 0.1) for all tests. After applying the BH correction, out of 176 tests and 39 significant occurrences, only nine times did the TE rating scores differed significantly from the NEP scores. Specifically, four differences were detected for fabric roughness, followed by two differences for isotropy, and one each for bumpiness, hardness, and stickiness. The ratings per textile swatch for each case are depicted through violin plots in Fig 4A–4H. For the sake of discernability of the large dataset collected, we present the data for each touch attribute separately in Fig 4A–4H. The thicker regions for each violin plot represent a higher number of responses given by the participants on a particular response anchor on the Likert scale. The dashed (-) line in Fig 4A–4H shows the mutual consensus of all textile experts on the Likert score rating of the respective textile swatch. For example, as shown in Fig 4A all experts assigned a score of 2 to fabrics “K3B,” “K6W” and “L2S” on a 5-point scale of hardness. No consensus was observed among the NEP data.

#### Hardness

The TE and NEP scored average fabric hardness similar to each other, except for the fabric code M4S, for which the TE scores differed significantly from the NEP scores (*Z* = 2.98; BH-adjusted p-value, *Q* = 0.002). All experts scored M4S as “4” on a scale of hardness, while the NEP ratings for the same varied between 2–4 with a median rating of 3. Additionally, as noted in Fig 4A, both TE and NEP perceived the mesh fabrics to be highest on the hardness scale, while the woven and knitted fabrics received the lowest hardness scores from TE and NEP. Despite the subjective idiosyncrasies involved in the human assessment of textile tactility, humans can have some sense of agreement in these assessments, as shown by our results and noted by other studies [8,34].

#### Roughness

The roughness ratings of TE differed significantly from those of NEP for four out of the 22 textile swatches. Of the four significant results, three were woven swatches: W3CH (*Z* = 3.67, *Q* = 0.0006), W6G (*Z* = 2.82, *Q* = 0.005), W9BW (*Z* = 2.97, *Q* = 0.003), and K3B (*Z* = 3.15, *Q* = 0.002). In all these cases TE scores of roughness were higher compared to the NEP. Similar to the hardness results, both the TE and NEP perceived the mesh fabrics to be the roughest across all fabric construction types (median = 4 in both groups). The laminated fabrics were perceived as smoothest by both TE_median_ = 2 and NEP_median_ = 1. The general trend of roughness data indicates that, overall, TE perceived higher roughness in textile swatches compared to NEP.

#### Bumpiness

Overall, bumpiness data exhibited a consensus between TE and NEP scores, except for a significant difference between the two groups observed for a rating of W3CH (*Z* = 2.94, *Q* = 0.003; TE_median_ = 4, NEP_median_ = 1). As laminated fabrics have inherently smooth surfaces compared to other fabric constructions, we anticipated that they would receive the lowest scores for bumpiness which was supported by our results (TE_median_ = 1.5 and NEP_median_ = 1). In contrast, mesh textile swatches received the highest scores for bumpiness, TE_median_ = 4, and NEP_median_ = 3. Interestingly on average, bumpiness of both knitted and woven (TE_median_ = 3 and NEP_median_ = 2) textiles was perceived alike.

#### Stickiness

The TE and NEP scored average fabric stickiness almost similar to each other, except for W3CH, for which the TE_median_ = 3 scores differed significantly (*Z* = 2.89, *Q* = 0.004) from the NEP_median_ = 1 scores. Additionally, as noted in Fig 4D, both TE (median = 3) and NEP (median = 3) perceived the mesh textiles to be highest on the scale of stickiness, followed by knitted (TE_median_ = 2, NEP_median_ = 2), woven (TE_median_ = 3, NEP_median_ = 2), and laminated (TE_median_ = 2, NEP_median_ = 2) textiles.

#### Scratchiness

The BH correction did not reveal any significant differences between the TE and NEP scratchiness scores for all textile swatches. Similar to the bumpiness, stickiness, and roughness results, the mesh textiles had the highest perceived scratchiness scores (TE_median_ = 4, NEP_median_ = 4). On the other hand, laminated textiles were perceived to be the lowest on the scale of scratchiness by both TE_median_ = 1.5 and NEP_median_ = 1. The knitted and woven textiles received a perceived scratchiness score of medians = 2 from both textile experts and non-experts.

#### Hairiness

Interestingly, textile experts and non-expert participants reached a consensus on the hairiness scores of all textile swatches. Overall, woven and knitted textile swatches received the highest scores (TE_median_ = 2, NEP_median_ = 2). As expected, both laminated and mesh textiles received the lowest hairiness scores from TE_median_ = 1 and NEP_median_ = 1. Overall, NEP rated knitted textiles higher on a scale of hairiness compared to textile experts; however, these differences were not statistically significant.

#### Uniformity

Similar to the scratchiness and hairiness data, after applying BH correction, we did not find any significant difference between the TE and NEP ratings for the uniformity of all textile swatches. The TE and NEP scores matched perfectly for the knitted (TE_median_ = 4; NEP_median_ = 4), mesh (TE_median_ = 4; NEP_median_ = 4), and woven (TE_median_ = 3; NEP_median_ = 3) textile swatches. For laminated fabrics, textile experts (median = 3), and non-expert participants (median = 4.5) perceived the uniformity of the swatches differently; however, this difference was not statistically significant.

#### Isotropy

As noted in Fig 4G and 4H), the TE and NEP scored the isotropy of textile swatches almost similar (*Z* = -3.27; *Q* = 0.001) to each other, except for W11N (TE_median_ = 1 and NEP_median_ = 3 scores). The NEP scores varied the most (although they were not found to be statistically significant) between the response anchors for uniformity and isotropy. We anticipated that these two variables would be the most difficult for the human assessment of textile touch perception.

### The predictive models of touch attribute ratings based on tactile sensitivity

**Fig 5:**
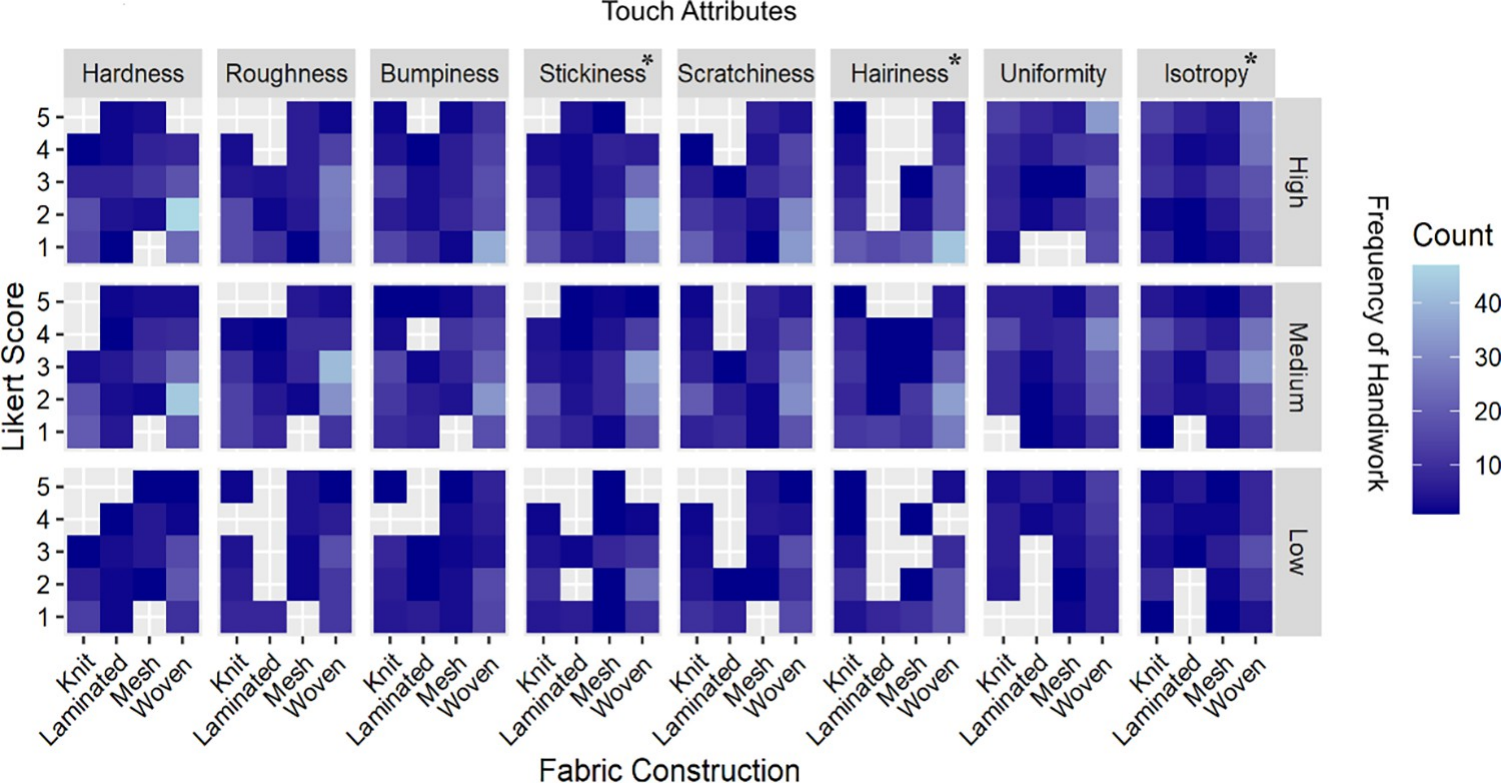
Heatmap indicating the effect of handiwork frequency (low 4, medium 8, high 8) on individual assessment of the eight touch attributes across all fabric construction types. The attribute with an asterisk shows significant differences among groups of individuals who perform high/medium/low handiwork. The darker blue shade depicts a lower response rate as opposed to the higher number of responses depicted by the sky blue color in the heatmap.

**Fig 6:**
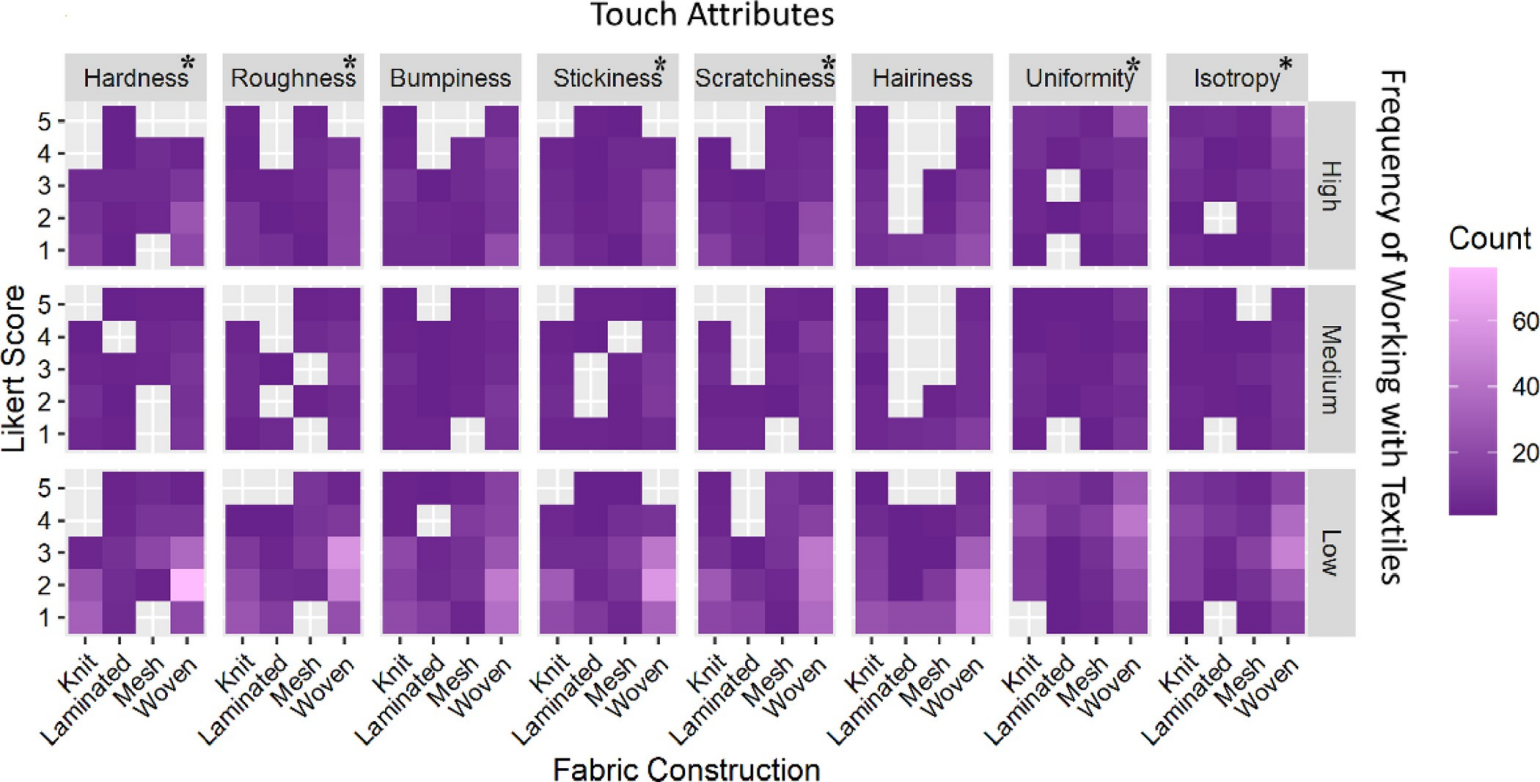
Heatmap indicating the effect of prior experience with textiles (low 10, medium 5, high 5) on individual assessment of the eight touch attributes across all fabric construction types. The attribute with an asterisk shows significant differences among groups of individuals who have high/medium/low prior experience working with textiles. The darker purple shade depicts a lower response rate as opposed to the higher number of responses depicted by the pink color in the heatmap.

**Fig 7:**
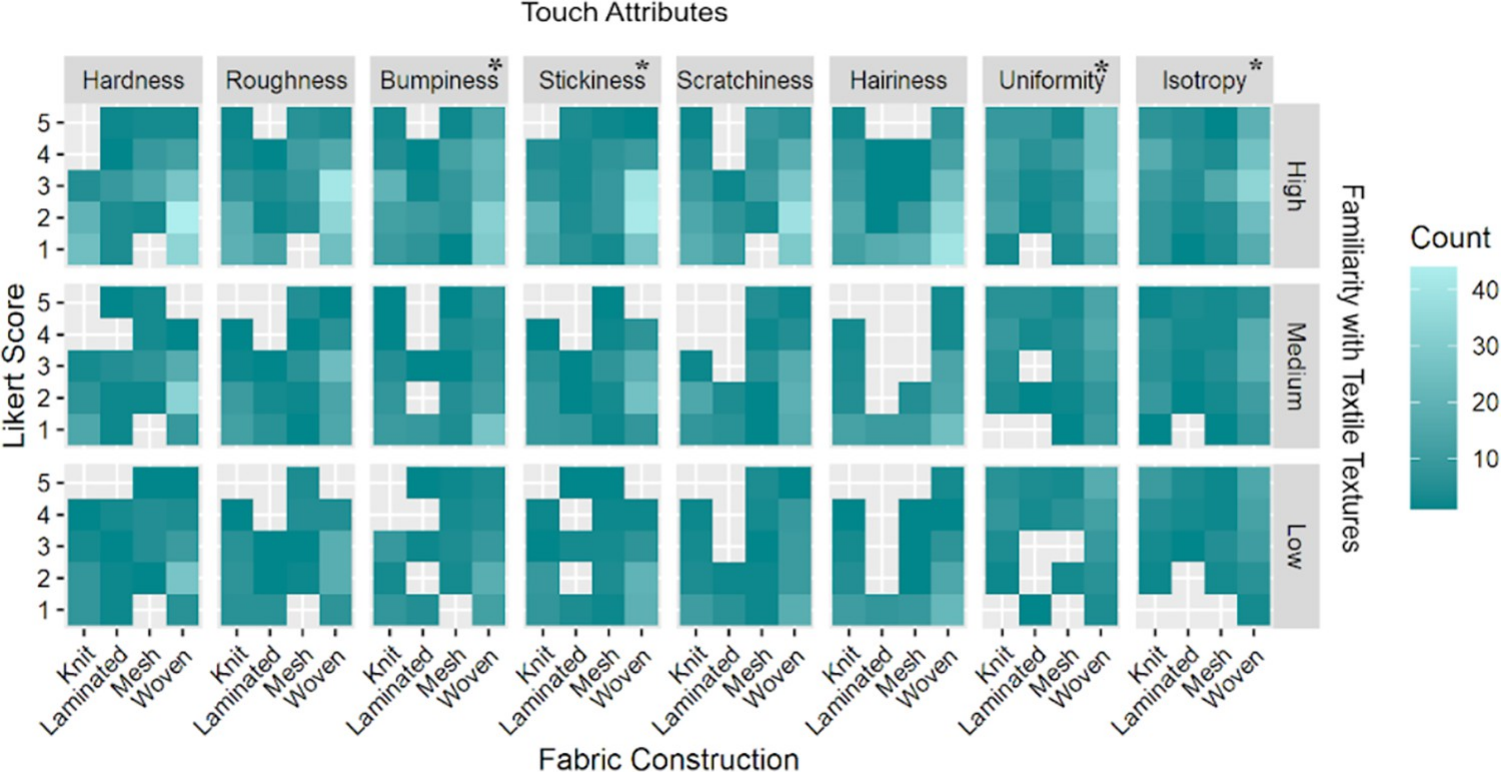
Heatmap indicating the effect of the knowledge of textile textures (low 5, medium 5, high 10) on individual assessment of the eight touch attributes across all fabric construction types. The attribute with an asterisk shows significant differences among groups of individuals who have high/medium/low prior experience working with textiles. The darker cyan-green shade depicts a lower response rate as opposed to the higher number of responses depicted by the seafoam green color in the heatmap.

We predicted that the evaluation of the eight touch attributes would be different for participants with higher familiarity and experience with different textured objects, including textiles. Therefore, we assessed tactile sensitivity based on three factors; “frequency of handiwork” (Fig 5); “frequency of working with textiles” (Fig 6), and “familiarity with textile textures” (Fig 7), in addition to fabric construction on the NEP scores for eight touch attributes using an ordinal logistic regression model. In the pre-experimental survey, the participants were asked to indicate their frequency of engaging in handiwork on a 5-point Likert scale, which was later converted into low (1–2), medium (3), or high (4–5) scores. All combinations of significant effects of tactile sensitivity on touch attributes with the basic frequency statistics are listed in S2–S4 Tables under the supplementary information of this paper. In addition, we included the effects of fabric construction in the models. For all eight touch attributes, the NEP scores differed significantly (*Q*<0.001) by varied fabric construction, matching the highest LogWorth of construction in each of the eight models (*LogWorth* ranged from 4.04 to 26.7 for the eight attributes).

#### Frequency of handiwork

For this study, we defined handiwork as, “Any object or art produced by hand such as hand embroidery, pottery, woodwork, and other crafts, etc.” As depicted in Fig 5, the frequency of performing handiwork significantly affected NEP scores for three attributes namely, hairiness (*LogWorth* 1.94, *Q* = 0.05), stickiness (*LogWorth* = 1.94, *Q* = 0.05), and isotropy (*LogWorth* = 1.29; *Q* = 0.07). The individuals who reported performing high amounts of handiwork rated the stickiness and hairiness at the lowest tail end of a 5-point Likert score compared to individuals who reported performing medium and low levels of handiwork. These individuals rated the textile isotropy attribute significantly higher (majority ratings between 5 and 4) compared to those who did not perform handiwork on a regular basis.

Interestingly, in the case of stickiness, participants involved in high-frequency handiwork scored the majority of textile swatches somewhat equally between 1 (31.82% of all fabrics) and 2 (34.09%) on a 5-point Likert scale. However, individuals with medium and low engagement with handiwork had majority ratings concentrated between 2 and 3 (S1 Table).

A similar trend was observed in hairiness scores. The participants who perform high-frequency handiwork rated hairiness relatively strongly at the lowest end of the Likert scale (1) (55.68%) than expected compared to those in medium (35.23%) and low (45.45%) handiwork performing groups (S1 Table). On the contrary, the attribute of isotropy was rated highest (5) by participants who performed high (28.41%) frequency of handiwork compared to those who performed medium (3 and 4; ~31%), or low (3; 30.68%) handiwork.

To put simply, the individuals who performed a high frequency of handiwork perceived lower levels of stickiness and hairiness while a higher level of isotropy among the set of textile swatches compared to their counterparts who performed handiwork less frequently. These findings show that tactile sensitivity varies among individuals who perform different levels of handiwork.

#### Frequency of working with textiles

Additionally, our curiosity extended to explore the potential impact of the “frequency of working with textiles” on NEP scores for the eight touch attributes. As noted in Fig 6, the frequency of regularly working with textiles did affect the participant ratings significantly for six touch attributes including roughness (*LogWorth* 2.32, *Q* = 0.05), isotropy (*LogWorth* 8.25, *Q* = 0.02), hardness (*LogWorth* 1.80, *Q* = 0.06), stickiness (*LogWorth* 3.51, *Q* = 0.04), scratchiness (*LogWorth* 3.21, *Q* = 0.04), and uniformity (*LogWorth* 6.75, *Q* = 0.02). However, hairiness and bumpiness ratings were not significantly different among the three groups of individuals who had prior experience working with textiles. The participants who frequently worked with textiles scored isotropy (31.82%) and uniformity (39.09%) at the highest (5) on a 5-point Likert scale. However, their responses were concentrated on the lower end of the Likert scale for scratchiness (1), stickiness (1 and 2), and roughness (1). Conversely, participants who worked less frequently with textiles had neutral opinions (Likert score 3) about isotropy (31.06%), uniformity (33.71%), and roughness (31.82%).

Our results indicated that individuals who work with textiles more often than others have a higher amount of sensitivity for uniformity and isotropy attributes while their Likert scores reflected lesser sensitivity for scratchiness, stickiness, and roughness. In other words, their scores lied more on the extreme ends for the majority of the tactile attributes. On the other hand, the individuals with lesser experience with textiles remained neutral on their scores for most of the tactile attributes. Another interesting observation as seen in Fig 5. is that individuals with the least amount of experience working with textiles rated all the attributes (except uniformity) between 1–3 on a 5-point Likert scale for the majority of the textile swatches. While there can be several reasons for these results, our observation during data collection was that individuals who often work with textiles were more confident in textural ratings of the textile swatches. This could be credited to their prior experience and familiarity with textile textures.

#### Familiarity with textile textures

As can be seen in Fig 7, participants with high familiarity/knowledge of textile textures scored the textile swatches significantly different for bumpiness (*LogWorth* 1.50, *Q* = 0.06), isotropy (*LogWorth* 8.61, *Q* = 0.02), stickiness (*LogWorth* 1.70, *Q* = 0.06), and uniformity (*LogWorth* 4.27, *Q* = 0.03) compared to those who had low familiarity with textile textures.

A statistically significant difference was observed among the individuals with high/medium/low familiarity with textile textures. The bumpiness scores of participants with low prior familiarity were fairly equally distributed between 1, 2, and 3 on a 5-point Likert scale (ranging from 28.41 to 27.27%). Whereas the majority of the individuals with high familiarity with textile textures rated bumpiness as 2 (27.73%). A rather surprisingly high frequency (45.45%) of individuals with medium familiarity rated bumpiness of textile swatches as 1. We observed interesting trends in the stickiness data; individuals with both medium (35.45%) and high (34.09%) familiarity with textile textures scored stickiness at 2. However, the majority of the individuals with low familiarity with textile textures were torn between stickiness 1 (37.00%) and 2 (37.00%).

A prior familiarity with textile textures also significantly affected the ratings of the isotropy attribute. The participants with low familiarity with textile textures scored the isotropy (5; 35.23%) higher compared to those with high (3; 29.55%) and medium (3; 28.18 and 4; 27.27) familiarity with textile textures. A similar trend was observed in the uniformity ratings as well. It is notable that even though some individuals have limited experience working with textiles they were able to rate both isotropy and uniformity closer to each other. Although these two attributes are perceived as similar to each other, the two differ as well. Uniformity is often associated with homogeneity while isotropy is associated with the homogeneity with directionality of an attribute.

### Correlations among tactile attributes

**Fig 8:**
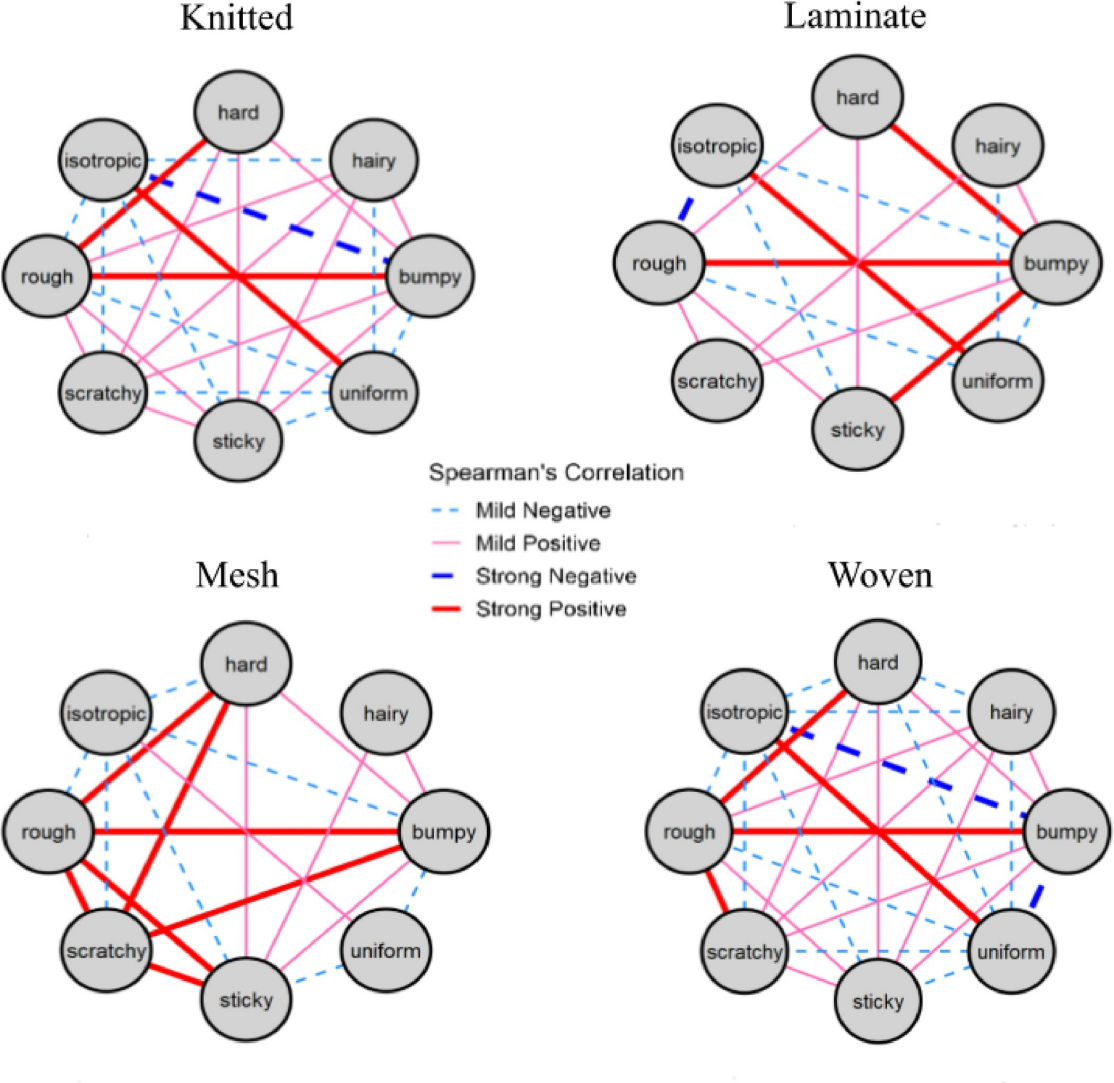
Correlation wheels for individual perceptual estimates of all tactile attributes. Only significant correlations after BH adjustment with FDR 0.1 were plotted. The solid red line indicates a strong positive relation, while the solid pink line depicts a mild positive correlation. The thick navy blue dashed line indicates a strong negative and the light blue dashed line depicts mild negative correlations between the tactile attributes as rated by the non-expert participants.

We studied the correlation among 8 touch attributes (hardness, roughness, bumpiness, stickiness, scratchiness, hairiness, uniformity, and isotropy) based on the fabric construction (knitted, laminated, mesh, and woven) variable using Spearman’s rank-order correlation Rho (R(s)) test depicted in Fig 8A–8D. To all 112 correlations we further applied BH correction at a FDR of 0.1 to find adjusted significant correlations and the associated strength. After the BH correction, a total of 90 correlations were found significant ranging from -0.61 to -0.16 on the negative correlation side and from 0.20 to 0.78 on the positive correlation. Since everything closer to zero on either side (between -0.16 and 0.2) would be “extremely weak” or no correlation as per BH correction, the -0.16 and 0.2 represented the thresholds of mild significance governed by the data. The mild positive correlations had R(s) between 0.2 and 0.5 and mild negative correlations had R(s) between -0.5 and -0.16. The strong positive correlations had a R(s) >0.5 while the strong negative (R(s)< -0.5), which is the opposite of it. A strong positive correlation was found between the tactile ratings of roughness and bumpiness attributes across all fabric constructions. On the other hand, isotropy was found to have a strong negative correlation with roughness for laminated and bumpiness for woven and knitted fabrics.

#### Knitted textile swatches

As noted in Fig 8A several positive correlations of varying strengths were observed among the eight attributes in knitted swatches. Roughness was strongly correlated with bumpiness and hardness, while isotropy had a strong positive correlation with uniformity. It also had a strong negative correlation with bumpiness. Both stickiness and scratchiness were found to have a mild positive correlation with bumpiness, hairiness, hardness, roughness, and with each other. Whereas uniformity had mild negative correlations with bumpiness, roughness, scratchiness, stickiness, and hairiness. The attribute of hardness did not share any correlation with isotropy, uniformity, and hairiness.

#### Laminated textile swatches

The correlations among the touch attributes of laminated textiles are depicted in Fig 8B. Bumpiness had a strong positive correlation with hardness, roughness, and stickiness. It also had a mild positive correlation with hairiness and scratchiness. As expected, it was found to be negatively correlated with both isotropy and uniformity. Similar to knitted textiles’ results, isotropy had a strong positive correlation with uniformity and a strong negative correlation with the roughness of the laminated textiles.

#### Mesh textile swatches

The mesh textiles had the greatest number of strong positive correlations of all fabric construction types. However, there were no strong negative correlations observed among the eight attributes of the mesh textiles. Both roughness and scratchiness were found to have strong positive correlations with stickiness, bumpiness, hardness, and with each other (Fig 7C). Similarly, bumpiness and stickiness had a mild positive correlation with hairiness, hardness, and with each other. The attribute isotropy had a mild positive correlation with uniformity and a mild negative correlation with stickiness, roughness, scratchiness, hardness, and bumpiness.

#### Woven textile swatches

Most interestingly, all the attributes for woven fabrics had either a positive or a negative correlation of varying effect sizes with each other (Fig 8D). Roughness had a strong positive correlation with hardness, bumpiness, and scratchiness and a mild positive correlation with both hairiness and stickiness. Isotropy and uniformity had a strong positive correlation like knitted and laminated textiles. Both of these attributes also had a strong negative correlation with bumpiness and a mild negative correlation with roughness, scratchiness, stickiness, hairiness, and hardness.

## Discussion

Tactile feedback is a crucial asset in designing tangible virtual and real interfaces [35–38]. Through this information, consumer experiences and interaction with digitally presented merchandise can be both more intuitive and accessible. Researchers have created several haptic experiences, especially with textile-like materials [39–43]. However, to date, a deeper understanding of how the mechanical behaviors of viscoelastic materials such as textiles affect human touch perception is not fully known. We designed this study to deeply explore the subject of textile touch perception and discuss our main findings in light of the existing literature below:

Firstly, in a contrast evaluation of the TE and NEP ratings of eight touch attributes across all 22 textile swatches (176 comparisons total), we found that only nine times the TE ratings differed significantly from the NEP ratings. This shows that despite the subjectivity involved in the human tactile perception of textile textures, the textile experts and lay consumers rated the tactile assessment of swatches statistically similar to each other. Our finding is supported by several other studies [8,34,44,45] in the field of textile touch perception. Further looking at the distribution of 9 significant differences among fabric construction types, it was interesting to observe that eight of the nine (four roughness, two isotropic, one bumpiness, and one stickiness) significant differences were found among the tactile assessment of woven textiles. However, only one significant difference was observed in the hardness ratings of the mesh textiles. In other words, there were no statistically significant differences between the TE and NEP ratings of knitted and laminated textiles.

From an individual touch attribute standpoint, the previously conducted studies [8,45], concluded that all participants agreed on the roughness and softness ratings of the fabrics presented to them. Unlike these studies [8,45], we included textile experts’ ratings of touch attributes in our study. On the other hand, our results showed that the roughness attribute had the highest disagreement among assessors (four out of nine significant differences). Since the textile experts hold high knowledge and experience working with textiles their tactile sensitivity is different compared to the non-expert participants. This shows that subject matter (textiles) expertise makes a statistically significant difference in how we perceive fabric roughness. However, we didn’t observe this trend for other touch attributes in our data.

To further understand how tactile sensitivity affected the eight touch attributes ratings, we took a deeper dive at the distribution of NEP ratings (based on three factors: engagement with textiles, other handiwork, and prior knowledge of textile textures) in a logistic regression model analysis (Figs 5–7). We found that participants who rated themselves as high on these three factors assessed the isotropy and stickiness attributes of the textile swatches significantly different from those at the low end of the spectrum. Moreover, those who frequently engaged with textiles rated all the attributes significantly different from others, except for bumpiness and hairiness. This further supported our findings above that tactile sensitivity affects human touch perception of textile surfaces. We observed a general trend that the assessors who self-rated themselves as “high” on 3 tactile sensitivity factors rated the touch attributes on the extreme ends of the Likert scale, perhaps demonstrating more confidence in their tactile attribute ratings and reflecting higher tactile sensitivity to textural surfaces. To our knowledge, Dillon et al., 2001 [46] is the only study that recommended including textile subject experts in textile touch perception research. Although, they did not include textile subject experts in their study. We addressed this gap by comparing (a) TE and NEP ratings of tactile assessment and (b) further comparing the tactile ratings of NEP based on their levels of interaction and knowledge of textural surfaces. Several studies [8,10–12,15] in the field recruited lay consumer participants for the textile touch perception assessment and examined the effects of demographic factors such as gender, age, race/ethnicity, and geographical background on tactile ratings of the participants.

Lastly, we were interested in exploring empirical data-based correlation between eight touch attributes. We found positive correlations of varying strength between tactile ratings of roughness, bumpiness, scratchiness, hardness, and hairiness across all fabric constructions. Our findings align with Degraen et al., 2021 [36] who found similar observations in their study except they found hairiness to be positively correlated with uniformity and isotropy whilst negatively correlated with the rest of the attributes. Our results also revealed a positive correlation between isotropy and uniformity for woven, knitted, laminated, and mesh textiles. Isotropy and uniformity did not have any positive correlation with the rest of the attributes. Interestingly, hairiness didn’t have any correlation with hardness except for a mild negative correlation among woven textile swatches.

## Conclusion

In the realm of textile haptics, the experience of perceiving textures through touch entails a sophisticated interaction among the skin’s mechanoreceptors, neural pathways, and higher brain regions responsible for processing and synthesizing tactile input. This intricate mechanism enables humans to discern and enjoy the varied and distinct sensations linked to diverse textile surfaces [47]. Through touch, humans rely on mechanoreceptors in their skin to detect the physical properties of materials, including roughness, thickness, uniformity, and other geometrical variables that makeup “textured” feeling of a fabric’s surface [8,47]. The findings of our study revealed that the tactile sensitivity affects the subjective assessment of this complex phenomenon up to some extent. Additionally, the assessment of isotropy and stickiness were consistently affected by assessors’ high prior knowledge of and engagement with textiles and other handiworks. Moreover, major differences were observed between TE and NEP roughness ratings of (4 of the 12) woven textile swatches. This shows that roughness assessment of woven textiles was significantly impacted by the subject knowledge and experience of the assessor. Our perception of a material’s textures is significantly influenced by its roughness and coarseness [3,24,37,43]. Roughness being such an important attribute in forming the overall judgement of material’s touch perception must be further studied. Additional analysis of interrelations among the touch attributes revealed that isotropy and uniformity were positively correlated with each other while these two had an overall negative correlation with the other six attributes across all fabric construction types. The intricacies of human touch interaction (with textile surfaces) presented in this study can make significant contributions to the fields of machine learning-based assessment of fabric’s mechanical properties, design of haptic devices, augmented and virtual reality, and e-commerce presentation of textile merchandise. In the future works, we plan to develop innovative biomedical/therapeutic products for people with tactile impairment and challenges such as attention deficit hyperactivity disorder (ADHD), and autism spectrum disorder (ASD).

## Supporting information

S1 Table Levene test of homogeneity of variance between textile experts (TE) and non-expert participants (NEP) for each textile attribute across 22 textile swatches. It depicts that standard deviations (SD) are homogenous in five attributes and are not homogenous in three attributes (isotropy, scratchiness and stickiness) at alpha 0.05. Markedly, for scratchiness and stickiness, the SDs among textile experts (n = 5) are smaller than NEP (n = 20) in spite their smaller sample size.
(DOCX)

S2 Table Contingency tables demonstrating the association of frequency of engaging in handiwork (low, medium high) for stickiness (a), hairiness (b) and isotropy (c) attributes (Likert Scale 1 to 5), corresponding to Fig 5 in the paper. Each cell in the contingency table represents cumulative data across all types of fabric construction.
(DOCX)

S3 Table Contingency tables demonstrating the association of frequency of engaging with textiles (low, medium high) for hardness (a), roughness (b), stickiness (c), scratchiness (d), uniformity (e), and isotropy (f) attributes (Likert Scale 1 to 5), corresponding to Fig 6 in the paper. Each cell in the contingency table represents cumulative data across all types of fabric construction.
(DOCX)

S4 Table Contingency tables demonstrating the association of familiarity of textile textures (low, medium, high) for bumpiness (a), stickiness (b), uniformity (c), and isotropy (d) attributes (Likert Scale 1 to 5), corresponding to Fig 7 in the paper. Each cell in the contingency table represents cumulative data across all types of fabric construction.
(DOCX)
