# Supplementary material for: Tactile sensitivity alters textile touch perception
Source: PLoS One. 2024 Sep 18;19(9):e0308957. doi: 10.1371/journal.pone.0308957 (PMC11410198; doi:10.1371/journal.pone.0308957)
Supplement: S1 Table — It depicts that standard deviations (SD) are homogenous in five attributes and are not homogenous in three attributes (isotropy, scratchiness and stickiness) at alpha 0.05. Markedly, for scratchiness and stickiness, the SDs among textile experts (n = 5) are smaller than NEP (n = 20) in spite their smaller sample size. (DOCX) [file pone.0308957.s001.docx]

**S1 Table.** Levene test of homogeneity of variance between textile experts (TE) and non-expert participants (NEP) for each textile attribute across 22 textile swatches. It depicts that standard deviations (SD) are homogenous in five attributes and are not homogenous in three attributes (isotropy, scratchiness and stickiness) at alpha 0.05. Markedly, for scratchiness and stickiness, the SDs among textile experts (n=5) are smaller than NEP (n=20) in spite their smaller sample size.

| **Attribute** | **F Ratio** | **DFNum^a^** | **DFDen^b^** | **p-Value** | **TE SD** | **NEP SD** | **Power^c^** |
| --- | --- | --- | --- | --- | --- | --- | --- |
| Bumpiness | 0.351046 | 1 | 548 | 0.553766 | 1.235052 | 1.276999 | 76.96% |
| Hairiness | 0.429756 | 1 | 548 | 0.512384 | 1.145212 | 1.123038 | 83.22% |
| Hardness | 0.582597 | 1 | 548 | 0.445625 | 1.105751 | 1.050375 | 85.63% |
| Isotropy | 19.85602 | 1 | 548 | 0.00001 | 1.502958 | 1.22983 | 61.79% |
| Roughness | 1.03382 | 1 | 548 | 0.309712 | 1.102011 | 1.149516 | 85.76% |
| Scratchiness | 11.74027 | 1 | 548 | 0.000658 | 0.979566 | 1.220383 | 91.68% |
| Stickiness | 12.14544 | 1 | 548 | 0.000531 | 0.84514 | 1.034065 | 97.09% |
| Uniformity | 1.560494 | 1 | 548 | 0.212126 | 1.38709 | 1.298802 | 67.89% |

**^a, b^** Degrees of freedom for the numerator and denominator, respectively.

**^c^** Statistical power for two independent sample means, using alpha of 0.05, listed SDs of each group, n=5 for TE and n=20 for NEP, and the delta (difference in Likert scale to detect) of 2.
