# Supplementary material for: Tactile sensitivity alters textile touch perception
Source: PLoS One. 2024 Sep 18;19(9):e0308957. doi: 10.1371/journal.pone.0308957 (PMC11410198; doi:10.1371/journal.pone.0308957)
Supplement: S2 Table — Each cell in the contingency table represents cumulative data across all types of fabric construction. (DOCX) [file pone.0308957.s002.docx]

**S2 Table.** Contingency tables demonstrating the association of frequency of engaging in

handiwork (low, medium high) for stickiness (a), hairiness (b) and isotropy (c) attributes (Likert Scale 1 to 5), corresponding to **Figure 5** in the paper. Each cell in the contingency table represents cumulative data across all types of fabric construction.

1. **stickiness**

| Count Total % Col % Row % Expected  Cell Chi^2 | 1 | 2 | 3 | 4 | 5 | Total |
| --- | --- | --- | --- | --- | --- | --- |
| Low | 22  5.00  18.80  25.00  23.4  0.0838 | 35  7.95  22.58  39.77  31  0.5161 | 25  5.68  22.12  28.41  22.6  0.2549 | 5  1.14  11.11  5.68  9  1.7778 | 1  0.23  10.00  1.14  2  0.5000 | 88  20.00 |
| Medium | 39  8.86  33.33  22.16  46.8  1.3000 | 60  13.64  38.71  34.09  62  0.0645 | 51  11.59  45.13  28.98  45.2  0.7442 | 22  5.00  48.89  12.50  18  0.8889 | 4  0.91  40.00  2.27  4  0.0000 | 176  40.00 |
| High | 56  12.73  47.86  31.82  46.8  1.8085 | 60  13.64  38.71  34.09  62  0.0645 | 37  8.41  32.74  21.02  45.2  1.4876 | 18  4.09  40.00  10.23  18  0.0000 | 5  1.14  50.00  2.84  4  0.2500 | 176  40.00 |
| Total | 117  26.59 | 155  35.23 | 113  25.68 | 45  10.23 | 10  2.27 | 440 |

1. **hairiness**

| Count Total % Col % Row % Expected  Cell Chi^2 | 1 | 2 | 3 | 4 | 5 | Total |
| --- | --- | --- | --- | --- | --- | --- |
| Low | 40  9.09  20.00  45.45  40  0.0000 | 29  6.59  24.58  32.95  23.6  1.2356 | 13  2.95  17.81  14.77  14.6  0.1753 | 2  0.45  6.25  2.27  6.4  3.0250 | 4  0.91  23.53  4.55  3.4  0.1059 | 88  20.00 |
| Medium | 62  14.09  31.00  35.23  80  4.0500 | 56  12.73  47.46  31.82  47.2  1.6407 | 34  7.73  46.58  19.32  29.2  0.7890 | 18  4.09  56.25  10.23  12.8  2.1125 | 6  1.36  35.29  3.41  6.8  0.0941 | 176  40.00 |
| High | 98  22.27  49.00  55.68  80  4.0500 | 33  7.50  27.97  18.75  47.2  4.2720 | 26  5.91  35.62  14.77  29.2  0.3507 | 12  2.73  37.50  6.82  12.8  0.0500 | 7  1.59  41.18  3.98  6.8  0.0059 | 176  40.00 |
| Total | 200  45.45 | 118  26.82 | 73  16.59 | 32  7.27 | 17  3.86 | 440 |

| Count Total % Col % Row % Expected  Cell Chi^2 | 1 | 2 | 3 | 4 | 5 | Total |
| --- | --- | --- | --- | --- | --- | --- |
| Low | 6  1.36  13.95  6.82  8.6  0.7860 | 22  5.00  28.21  25.00  15.6  2.6256 | 27  6.14  21.60  30.68  25  0.1600 | 17  3.86  15.32  19.32  22.2  1.2180 | 16  3.64  19.28  18.18  16.6  0.0217 | 88  20.00 |
| Medium | 15  3.41  34.88  8.52  17.2  0.2814 | 33  7.50  42.31  18.75  31.2  0.1038 | 55  12.50  44.00  31.25  50  0.5000 | 56  12.73  50.45  31.82  44.4  3.0306 | 17  3.86  20.48  9.66  33.2  7.9048 | 176  40.00 |
| High | 22  5.00  51.16  12.50  17.2  1.3395 | 23  5.23  29.49  13.07  31.2  2.1551 | 43  9.77  34.40  24.43  50  0.9800 | 38  8.64  34.23  21.59  44.4  0.9225 | 50  11.36  60.24  28.41  33.2  8.5012 | 176  40.00 |
| Total | 43  9.77 | 78  17.73 | 125  28.41 | 111  25.23 | 83  18.86 | 440 |

1. **isotropy**
