# Supplementary material for: Tactile sensitivity alters textile touch perception
Source: PLoS One. 2024 Sep 18;19(9):e0308957. doi: 10.1371/journal.pone.0308957 (PMC11410198; doi:10.1371/journal.pone.0308957)
Supplement: S3 Table — Each cell in the contingency table represents cumulative data across all types of fabric construction. (DOCX) [file pone.0308957.s003.docx]

**S3 Table.** Contingency tables demonstrating the association of frequency of engaging with textiles (low, medium high) for hardness (a), roughness (b), stickiness (c), scratchiness (d),

uniformity (e), and isotropy (f) attributes (Likert Scale 1 to 5), corresponding to **Figure 6** in the paper. Each cell in the contingency table represents cumulative data across all types of fabric construction.

1. **hardness**

| Count Total % Col % Row % Expected  Cell Chi^2 | 1 | 2 | 3 | 4 | 5 | Total |
| --- | --- | --- | --- | --- | --- | --- |
| Low | 57  12.95  53.77  21.59  63.6  0.6849 | 109  24.77  66.06  41.29  99  1.0101 | 66  15.00  60.00  25.00  66  0.0000 | 23  5.23  52.27  8.71  26.4  0.4379 | 9  2.05  60.00  3.41  9  0.0000 | 264  60.00 |
| Medium | 16  3.64  15.09  24.24  15.9  0.0006 | 16  3.64  9.70  24.24  24.75  3.0934 | 18  4.09  16.36  27.27  16.5  0.1364 | 11  2.50  25.00  16.67  6.6  2.9333 | 5  1.14  33.33  7.58  2.25  3.3611 | 66  15.00 |
| High | 33  7.50  31.13  30.00  26.5  1.5943 | 40  9.09  24.24  36.36  41.25  0.0379 | 26  5.91  23.64  23.64  27.5  0.0818 | 10  2.27  22.73  9.09  11  0.0909 | 1  0.23  6.67  0.91  3.75  2.0167 | 110  25.00 |
| Total | 106  24.09 | 165  37.50 | 110  25.00 | 44  10.00 | 15  3.41 | 440 |

1. **roughness**

| Count Total % Col % Row % Expected  Cell Chi^2 | 1 | 2 | 3 | 4 | 5 | Total |
| --- | --- | --- | --- | --- | --- | --- |
| Low | 63  14.32  55.75  23.86  67.8  0.3398 | 81  18.41  65.85  30.68  73.8  0.7024 | 84  19.09  66.14  31.82  76.2  0.7984 | 23  5.23  42.59  8.71  32.4  2.7272 | 13  2.95  56.52  4.92  13.8  0.0464 | 264  60.00 |
| Medium | 14  3.18  12.39  21.21  16.95  0.5134 | 12  2.73  9.76  18.18  18.45  2.2549 | 19  4.32  14.96  28.79  19.05  0.0001 | 16  3.64  29.63  24.24  8.1  7.7049 | 5  1.14  21.74  7.58  3.45  0.6964 | 66  15.00 |
| High | 36  8.18  31.86  32.73  28.25  2.1261 | 30  6.82  24.39  27.27  30.75  0.0183 | 24  5.45  18.90  21.82  31.75  1.8917 | 15  3.41  27.78  13.64  13.5  0.1667 | 5  1.14  21.74  4.55  5.75  0.0978 | 110  25.00 |
| Total | 113  25.68 | 123  27.95 | 127  28.86 | 54  12.27 | 23  5.23 | 440 |

1. **stickiness**

| Count Total % Col % Row % Expected  Cell Chi^2 | 1 | 2 | 3 | 4 | 5 | Total |
| --- | --- | --- | --- | --- | --- | --- |
| Low | 70  15.91  59.83  26.52  70.2  0.0006 | 102  23.18  65.81  38.64  93  0.8710 | 69  15.68  61.06  26.14  67.8  0.0212 | 21  4.77  46.67  7.95  27  1.3333 | 2  0.45  20.00  0.76  6  2.6667 | 264  60.00 |
| Medium | 13  2.95  11.11  19.70  17.55  1.1796 | 20  4.55  12.90  30.30  23.25  0.4543 | 18  4.09  15.93  27.27  16.95  0.0650 | 10  2.27  22.22  15.15  6.75  1.5648 | 5  1.14  50.00  7.58  1.5  8.1667 | 66  15.00 |
| High | 34  7.73  29.06  30.91  29.25  0.7714 | 33  7.50  21.29  30.00  38.75  0.8532 | 26  5.91  23.01  23.64  28.25  0.1792 | 14  3.18  31.11  12.73  11.25  0.6722 | 3  0.68  30.00  2.73  2.5  0.1000 | 110  25.00 |
| Total | 117  26.59 | 155  35.23 | 113  25.68 | 45  10.23 | 10  2.27 | 440 |

1. **scratchiness**

| Count Total % Col % Row % Expected  Cell Chi^2 | 1 | 2 | 3 | 4 | 5 | Total |
| --- | --- | --- | --- | --- | --- | --- |
| Low | 73  16.59  54.89  27.65  79.8  0.5794 | 82  18.64  64.06  31.06  76.8  0.3521 | 62  14.09  65.96  23.48  56.4  0.5560 | 29  6.59  51.79  10.98  33.6  0.6298 | 18  4.09  62.07  6.82  17.4  0.0207 | 264  60.00 |
| Medium | 15  3.41  11.28  22.73  19.95  1.2282 | 13  2.95  10.16  19.70  19.2  2.0021 | 17  3.86  18.09  25.76  14.1  0.5965 | 16  3.64  28.57  24.24  8.4  6.8762 | 5  1.14  17.24  7.58  4.35  0.0971 | 66  15.00 |
| High | 45  10.23  33.83  40.91  33.25  4.1523 | 33  7.50  25.78  30.00  32  0.0313 | 15  3.41  15.96  13.64  23.5  3.0745 | 11  2.50  19.64  10.00  14  0.6429 | 6  1.36  20.69  5.45  7.25  0.2155 | 110  25.00 |
| Total | 133  30.23 | 128  29.09 | 94  21.36 | 56  12.73 | 29  6.59 | 440 |

1. **uniformity**

| Count Total % Col % Row % Expected  Cell Chi^2 | 1 | 2 | 3 | 4 | 5 | Total |
| --- | --- | --- | --- | --- | --- | --- |
| Low | 21  4.77  50.00  7.95  25.2  0.7000 | 43  9.77  54.43  16.29  47.4  0.4084 | 53  12.05  60.92  20.08  52.2  0.0123 | 89  20.23  74.17  33.71  72  4.0139 | 58  13.18  51.79  21.97  67.2  1.2595 | 264  60.00 |
| Medium | 13  2.95  30.95  19.70  6.3  7.1254 | 16  3.64  20.25  24.24  11.85  1.4534 | 19  4.32  21.84  28.79  13.05  2.7128 | 7  1.59  5.83  10.61  18  6.7222 | 11  2.50  9.82  16.67  16.8  2.0024 | 66  15.00 |
| High | 8  1.82  19.05  7.27  10.5  0.5952 | 20  4.55  25.32  18.18  19.75  0.0032 | 15  3.41  17.24  13.64  21.75  2.0948 | 24  5.45  20.00  21.82  30  1.2000 | 43  9.77  38.39  39.09  28  8.0357 | 110  25.00 |
| Total | 42  9.55 | 79  17.95 | 87  19.77 | 120  27.27 | 112  25.45 | 440 |

1. **isotropy**

| Count Total % Col % Row % Expected  Cell Chi^2 | 1 | 2 | 3 | 4 | 5 | Total |
| --- | --- | --- | --- | --- | --- | --- |
| Low | 18  4.09  41.86  6.82  25.8  2.3581 | 51  11.59  65.38  19.32  46.8  0.3769 | 82  18.64  65.60  31.06  75  0.6533 | 74  16.82  66.67  28.03  66.6  0.8222 | 39  8.86  46.99  14.77  49.8  2.3422 | 264  60.00 |
| Medium | 13  2.95  30.23  19.70  6.45  6.6516 | 16  3.64  20.51  24.24  11.7  1.5803 | 18  4.09  14.40  27.27  18.75  0.0300 | 10  2.27  9.01  15.15  16.65  2.6560 | 9  2.05  10.84  13.64  12.45  0.9560 | 66  15.00 |
| High | 12  2.73  27.91  10.91  10.75  0.1453 | 11  2.50  14.10  10.00  19.5  3.7051 | 25  5.68  20.00  22.73  31.25  1.2500 | 27  6.14  24.32  24.55  27.75  0.0203 | 35  7.95  42.17  31.82  20.75  9.7861 | 110  25.00 |
| Total | 43  9.77 | 78  17.73 | 125  28.41 | 111  25.23 | 83  18.86 | 440 |
