# Supplementary material for: Tactile sensitivity alters textile touch perception
Source: PLoS One. 2024 Sep 18;19(9):e0308957. doi: 10.1371/journal.pone.0308957 (PMC11410198; doi:10.1371/journal.pone.0308957)
Supplement: S4 Table — Each cell in the contingency table represents cumulative data across all types of fabric construction. (DOCX) [file pone.0308957.s004.docx]

**S4 Table.** Contingency tables demonstrating the association of familiarity of textile textures (low, medium, high) for bumpiness (a), stickiness (b), uniformity (c), and isotropy (d) attributes (Likert Scale 1 to 5), corresponding to **Figure 7** in the paper. Each cell in the contingency table represents cumulative data across all types of fabric construction.

1. **bumpiness**

| Count Total % Col % Row % Expected  Cell Chi^2 | 1 | 2 | 3 | 4 | 5 | Total |
| --- | --- | --- | --- | --- | --- | --- |
| Low | 25  5.98  20.33  28.41  25.8947  0.0309 | 24  5.74  22.02  27.27  22.9474  0.0483 | 24  5.74  26.67  27.27  18.9474  1.3474 | 8  1.91  13.56  9.09  12.4211  1.5736 | 7  1.67  18.92  7.95  7.78947  0.0800 | 88  21.05 |
| Medium | 50  11.96  40.65  45.45  32.3684  9.6042 | 24  5.74  22.02  21.82  28.6842  0.7649 | 15  3.59  16.67  13.64  23.6842  3.1842 | 11  2.63  18.64  10.00  15.5263  1.3195 | 10  2.39  27.03  9.09  9.73684  0.0071 | 110  26.32 |
| High | 48  11.48  39.02  21.82  64.7368  4.3271 | 61  14.59  55.96  27.73  57.3684  0.2299 | 51  12.20  56.67  23.18  47.3684  0.2784 | 40  9.57  67.80  18.18  31.0526  2.5781 | 20  4.78  54.05  9.09  19.4737  0.0142 | 220  52.63 |
| Total | 123  29.43 | 109  26.08 | 90  21.53 | 59  14.11 | 37  8.85 | 418 |

1. **stickiness**

| Count Total % Col % Row % Expected  Cell Chi^2 | 1 | 2 | 3 | 4 | 5 | Total |
| --- | --- | --- | --- | --- | --- | --- |
| Low | 33  7.89  29.20  37.50  23.7895  3.5660 | 33  7.89  22.45  37.50  30.9474  0.1361 | 14  3.35  13.08  15.91  22.5263  3.2273 | 6  1.44  14.63  6.82  8.63158  0.8023 | 2  0.48  20.00  2.27  2.10526  0.0053 | 88  21.05 |
| Medium | 27  6.46  23.89  24.55  29.7368  0.2519 | 39  9.33  26.53  35.45  38.6842  0.0026 | 33  7.89  30.84  30.00  28.1579  0.8327 | 10  2.39  24.39  9.09  10.7895  0.0578 | 1  0.24  10.00  0.91  2.63158  1.0116 | 110  26.32 |
| High | 53  12.68  46.90  24.09  59.4737  0.7047 | 75  17.94  51.02  34.09  77.3684  0.0725 | 60  14.35  56.07  27.27  56.3158  0.2410 | 25  5.98  60.98  11.36  21.5789  0.5424 | 7  1.67  70.00  3.18  5.26316  0.5732 | 220  52.63 |
| Total | 113  27.03 | 147  35.17 | 107  25.60 | 41  9.81 | 10  2.39 | 418 |

1. **uniformity**

| Count Total % Col % Row % Expected  Cell Chi^2 | 1 | 2 | 3 | 4 | 5 | Total |
| --- | --- | --- | --- | --- | --- | --- |
| Low | 5  1.20  12.50  5.68  8.42105  1.3898 | 11  2.63  14.86  12.50  15.5789  1.3458 | 13  3.11  15.85  14.77  17.2632  1.0528 | 29  6.94  24.79  32.95  24.6316  0.7747 | 30  7.18  28.57  34.09  22.1053  2.8195 | 88  21.05 |
| Medium | 11  2.63  27.50  10.00  10.5263  0.0213 | 15  3.59  20.27  13.64  19.4737  1.0277 | 22  5.26  26.83  20.00  21.5789  0.0082 | 33  7.89  28.21  30.00  30.7895  0.1587 | 29  6.94  27.62  26.36  27.6316  0.0678 | 110  26.32 |
| High | 24  5.74  60.00  10.91  21.0526  0.4126 | 48  11.48  64.86  21.82  38.9474  2.1041 | 47  11.24  57.32  21.36  43.1579  0.3420 | 55  13.16  47.01  25.00  61.5789  0.7029 | 46  11.00  43.81  20.91  55.2632  1.5527 | 220  52.63 |
| Total | 40  9.57 | 74  17.70 | 82  19.62 | 117  27.99 | 105  25.12 | 418 |

1. **isotropy**

| Count Total % Col % Row % Expected  Cell Chi^2 | 1 | 2 | 3 | 4 | 5 | Total |
| --- | --- | --- | --- | --- | --- | --- |
| Low | 3  0.72  6.98  3.41  9.05263  4.0468 | 11  2.63  14.67  12.50  15.7895  1.4528 | 20  4.78  17.24  22.73  24.4211  0.8004 | 23  5.50  21.70  26.14  22.3158  0.0210 | 31  7.42  39.74  35.23  16.4211  12.9435 | 88  21.05 |
| Medium | 10  2.39  23.26  9.09  11.3158  0.1530 | 24  5.74  32.00  21.82  19.7368  0.9208 | 31  7.42  26.72  28.18  30.5263  0.0074 | 30  7.18  28.30  27.27  27.8947  0.1589 | 15  3.59  19.23  13.64  20.5263  1.4879 | 110  26.32 |
| High | 30  7.18  69.77  13.64  22.6316  2.3990 | 40  9.57  53.33  18.18  39.4737  0.0070 | 65  15.55  56.03  29.55  61.0526  0.2552 | 53  12.68  50.00  24.09  55.7895  0.1395 | 32  7.66  41.03  14.55  41.0526  1.9962 | 220  52.63 |
| Total | 43  10.29 | 75  17.94 | 116  27.75 | 106  25.36 | 78  18.66 | 418 |
